# Bioconversion of sago processing wastewater into biodiesel: Optimization of lipid production by an oleaginous yeast, *Candida tropicalis* ASY2 and its transesterification process using response surface methodology

**DOI:** 10.1186/s12934-021-01655-7

**Published:** 2021-08-26

**Authors:** Kiruthika Thangavelu, Pugalendhi Sundararaju, Naganandhini Srinivasan, Sivakumar Uthandi

**Affiliations:** 1 Department of Renewable Energy Engineering, Agricultural Engineering College and Research Institute, Tamil Nadu Agricultural University, Coimbatore, 641 003 India; 2 Biocatalysts Laboratory, Department of Agricultural Microbiology, Tamil Nadu Agricultural University, Coimbatore, 641 003 Tamil Nadu India

**Keywords:** Sago processing wastewater, Yeast lipid, Transesterification, Biodiesel, Response surface methodology

## Abstract

**Background:**

Biodiesel is an eco-friendly and renewable energy source and a valuable substitute for petro-diesel. Sago processing wastewater (SWW), a by-product of the cassava processing industry, has starch content ranging from 4 to 7 g L^–1^ and serves as an outstanding source for producing microbial lipids by the oleaginous microorganisms. In the present study, *Candida tropicalis* ASY2 was employed to optimize single-cell oil (SCO) production using SWW and subsequent transesterification by response surface methodology. Variables such as starch content, yeast extract, airflow rate, pH, and temperature significantly influenced lipid production in a preliminary study. The lipid production was scaled up to 5 L capacity airlift bioreactor and its optimization was done by response surface methodology. The dried yeast biomass obtained under optimized conditions from 5 L bioreactor was subjected to a direct transesterification process. Biomass: methanol ratio, catalyst concentration, and time were the variables used to attain higher FAME yield in the transesterification optimization process.

**Results:**

Under optimized conditions, the highest lipid yield of 2.68 g L^–1^ was obtained with 15.33 g L^–1^ of starch content, 0.5 g L^–1^ of yeast extract, and 5.992 L min^–1^ of airflow rate in a bioreactor. The optimized direct transesterification process yielded a higher FAME yield of 86.56% at 1:20 biomass: methanol ratio, 0.4 M catalyst concentration, and a time of 6.85 h.

**Conclusions:**

Thus, this optimized process rendered the microbial lipids derived from *C. tropicalis* ASY2 as potentially alternative oil substitutes for sustainable biodiesel production to meet the rising energy demands.

**Supplementary Information:**

The online version contains supplementary material available at 10.1186/s12934-021-01655-7.

## Background

The interest in exploring new renewable biofuels as substitutes to petroleum-derived fuels has increased during the last several decades. Energy consumption in India accounts for 4% of global energy. India is the fifth-largest energy utilization nation after the USA, China, Russia, and Japan. As vehicle ownership has risen, the demand for petrol and diesel is anticipated to increase to 110 MMT (Million Metric Tons) and 31.1 MMT by 2021–2022, respectively [1].

Biodiesel, a green and clean fuel type, can be obtained from numerous feedstock like land-based crops, microbes, and waste grease comprising triacylglycerides or free fatty acids [2]. At present, 95% of the global biodiesel is made from plant oils like soybean, rapeseed, and palm oils [3]. Among these, the oil from soybean serves as a feedstock for biodiesel production on an industrial scale. However, it not only conflicts with food but also possesses several limitations such as land requirement, oxidative stability, and lesser oil yields [4]. Compared to oil floras, oleaginous microorganisms (OMs), such as microalgae, bacteria, yeast, and filamentous fungi, are considered alternative feedstock for lipids production from non-vegetable sources. They can accumulate more than 20% lipids (dry weight) and are extremely useful in biofuel production. They possess abundant benefits such as fast growth rate and oil productivity, reduced labor requirement, and lesser land coverage. Thus, they are feasible feedstock for oil production [5].

The microbial lipid production process costs more than the production cost of vegetable and animal oils; mainly, the carbon sources used in the fermentation account for a significant portion [6]. To date, the foremost carbon source used for lipid production is glucose or other equally catabolized compounds. Oleaginous yeasts are well known to convert simple sugars into microbial lipids [7, 8]. Various feedstock like lignocellulosic biomass [9, 10], industrial waste [11, 12], and different wastewater streams [13] have been investigated for yeast oil production.

Sago processing wastewater (SWW) can serve as an excellent low-cost substrate for microbial lipid production. During starch extraction from tapioca tuber, about 20,000 to 30,000 L of water is required per ton of sago, eventually producing the same quantity of wastewater. Moreover, with biodegradable starch, the content varies between 4 to 7 g L^–1^ [14]. While the SWW is starchy, it provides an outstanding nutritive source for microbial growth and development along with lipid accumulation under specific conditions.

At present, there is an enduring requisite to develop an effective, robust, and cost-economic process that utilizes SWW directly and transforms it into a valuable product like microbial lipids. The microbial oils are considered potential substitutes for biodiesel production due to the similarity in composition of their fatty acids with that of the vegetable oils. They mainly contain C16 and C18 fatty acids esterified in the form of triacylglycerols and possess a higher heating value than petro-diesel. If biodiesel is produced sustainably, it could replace about 27% of the diesel fuel in the transportation sector by 2050, and we can avoid 2.1 gigatonnes of CO_2_ emissions per year [15]. Previously, we isolated oleaginous yeast *Candida tropicalis* ASY2 from SWW for simultaneous decontamination and biodiesel production [16, 17]. In continuation with that, we characterized the yeast biomass for its bioenergy applications and determined its fuel properties [18].

The objective of the present work was to study the biodiesel production process experimentally and focusing on the optimization of lipid accumulation and direct transesterification process of the dry yeast biomass produced by the yeast strain of *C. tropicalis* ASY2 using SWW as a substrate. The optimization process was carried out using the Design-Expert software version 11.0. Response surface methodology (RSM) was applied to determine the optimal operating conditions for maximum lipid accumulation and FAME conversion efficiency. Furthermore, the interaction between the variables affecting lipid and biodiesel production was studied.

## Results and discussion

### Physicochemical characteristics of SWW

The values of physicochemical properties of SWW, such as pH, electrical conductivity (EC), starch, total solids (TS), total dissolved solids (TDS), total nitrogen (N), BOD, COD, cyanide, ammoniacal nitrogen, nitrate-nitrogen, and phosphate were 4.67, 6.30 dS m^−1^, 4.82 g L^–1^ [19], 4.57 g L^−1^, 4.16 g L^−1^, 0.54 g L^−1^, 5.04 g L^−1^, 70.67 g L^−1^, 4.46 mg L^–1^, 3.10 mg L^−1^, 5.48 mg L^−1^, and 611.67 mg L^−1^, respectively [17]. The important physicochemical properties of raw and treated SWW in ALB are given in Table 1.

**Table 1:** Physicochemical characteristics of raw and treated SWW in ALB

SWW Parameters	Raw SWW	Treated SWW in ALB
pH	4.67 (± 0.03)	6.5 (± 0.01)
BOD (g L^–1^)	5.04 (± 0.08)	1.60 (± 1.5)
COD (g L^–1^)	70.67 (± 0.06)	33.40 (± 0.84)
NO_3_ (mg L^–1^)	3.10 (± 0.02)	0.11 (± 0.005)
NH_4_ (mg L^–1^)	5.48 (± 0.05)	0.43 (± 0.01)
PO_4_ (mg L^–1^)	611.67 (± 0.01)	215.94 (± 0.61)
Cyanide (mg L^–1^)	4.46 (± 0.02)	1.74 (± 0.01)

BOD, Biological Oxygen Demand; COD, Chemical Oxygen Demand; NO_3_, Nitrate; NH_4_, Ammonium; PO_4_, Phosphate

## Investigation of influential factors for high lipid production by *C. tropicalis* ASY2 in SWW

### Effect of carbon sources

**Fig. 1:**
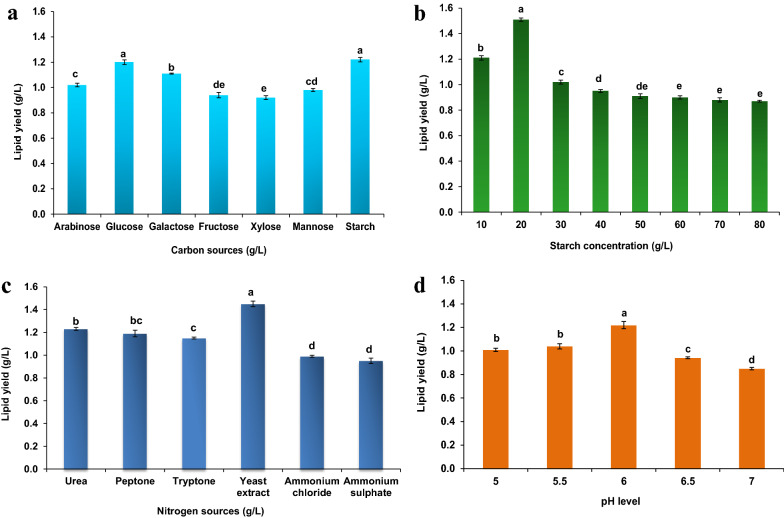
Study of different influential parameters on the lipid production of *C. tropicalis* ASY2. **a** Effect of different carbon sources, **b** Effect of starch concentrations from 10 to 80 g L^–1^, **c** Effect of different nitrogen sources **d** Effect of different pH. The experiment was conducted in a 250 mL Erlenmeyer flask (working volume: 50 mL) with a shaking speed of 150 rpm with different carbon and nitrogen sources, starch concentrations (10 to 80 g L^–1^), and pH levels (5 to 7). Values are mean (± standard error) (n = 3) and same alphabets in each column are not significantly different from each other within the observation day as determined by DMRT (*p* ≤ *0.05*)

Among different carbon sources tested, galactose, starch, and arabinose yielded a maximum biomass concentration of 2.65, 2.56, and 2.52 g L^–1^, respectively. However, a higher lipid yield was observed in starch (1.22 g L^–1^) followed by glucose (1.20 g L^–1^) and galactose (1.11 g L^–1^) (Fig. 1a). The highest lipid content of *C. tropicalis* ASY2 was reported in SWW enriched with glucose (48%) and starch (47.66%). Among the different sugars evaluated, amylase secretion was maximum in the starch-containing SWW (1.56 IU mL^–1^), indicating poly-sugar utilization by digestive enzymes. Mostly 80–87% of the sugars were utilized in most cases. Similar results were obtained when *R. glutinis* BCRC 22360 was grown in LCB hydrolysate [20], a lipid content of 48.23% using glucose concentration of 30 g L^–1^ was reported. Since the raw SWW already contains 4.82 g L^–1^ of starch, further experiments were continued with starch.

### Effect of starch concentrations

Among the different starch concentrations evaluated, a maximum lipid yield of 1.51 g L^–1^, lipid content of 51.19%, starch utilization of 89.54%, and amylase activity of 1.48 IU mL^–1^ was observed in 20 g L^–1^ of starch concentration. As the starch concentration increased after 20 to 80 g L^–1^, the lipid yield, biomass yield, and amylase secretion decreased gradually (Fig. 1b). It can be assumed that a high starch concentration resulting in a very gelatinized solution and limiting amylase diffusion can restrict the accessibility of glycosidic linkages [21, 22]. In our earlier study, the rheological study of SWW with varied starch concentrations (10 – 80 g L^−1^) also revealed that starch concentrations up to 20 g L^−1^ is best suited for microbial lipid production in airlift bioreactors [23]. Similarly, Ren H-Y, Liu B-F, Kong F, Zhao L and Ren N [24] reported that co-culture of microalgae *Scenedesmus* sp. and anaerobic sludge at 120 h produced a lipid yield of 0.36 g L^–1^ and 0.35 g L^–1^ at a starch concentration of 6 g L^–1^ and 10 g L^–1^ in starch wastewater. *C. terricola* JCM 24523, an oleaginous yeast at 240 h produced a maximum lipid content of 61.96% (lipid yield: 3.02 g L^–1^) and 43% on medium with 5% and 10% soluble starch, respectively [25]. Kraisintu P, Yongmanitchai W and Limtong S [26] reported that the yeast strain *Rhodosporidium toruloides* DMKU3-TK16 produced a lipid yield of 8.11 g L^–1^ and 7.95 g L^–1^ in a nitrogen-limited media (0.75 g L^–1^ yeast extract, 0.55 g L^–1^ (NH_4_)_2_SO_4_, pH: 6) containing a glucose concentration of 70 g L^–1^ and 90 g L^–1^, respectively. Thus, the differences in starch assimilation could be linked to distinct features of the species' metabolic systems [25].

### Effect of nitrogen sources

Among the different organic and inorganic sources evaluated, the organic nitrogen sources (urea, peptone, yeast extract, and tryptone) yielded higher biomass, lipid yield, and lipid content than inorganic nitrogen sources (Fig. 1c). Yeast extract yielded higher biomass of 3.15 g L^–1^ with a lipid content of 46.03%. Similarly, the amylase secretion and starch utilization differed in organic and inorganic nitrogen sources. The results agreed with those of Evans CT and Ratledge C [27], who observed that *R. toruloides* had much higher lipid accumulation when grown on organic nitrogen than inorganic nitrogen.

### Effect of pH

The pH of the fermentation medium is a significant environmental factor that affects the growth of cells and product formation [28]. There was no growth in the pH range of 3.5 to 5 when buffers of different pH were utilized to study the lipid yield and lipid content of *C. tropicalis* ASY2 in SWW (Fig. 1d). Some difference was observed in the lipid content under the pH range of 5.5–7, and higher lipid content was observed in pH 6 (47.66%) with an amylase secretion of 1.41 IU mL^–1^ and 85.76% starch utilization. Similarly, a maximum lipid production of 1.54 g L^–1^ was obtained at pH 6 when *Rhodotorula* sp. IIP-33 was grown in sugarcane bagasse hydrolysate [29].

### Effect of mineral nutrients

The addition of a mineral nutrient mixture resulted in a lipid yield of 1.39 g L^–1^ compared to the control (1.03 g L^–1^). The maximum starch utilization of 85.84% and amylase activity of 1.41 IU mL^–1^ were observed in a medium supplemented with a mineral nutrient mixture compared to SWW without the mineral nutrients (Table 2).

**Table 2:** Effect of mineral nutrients on the lipid yield of *C. tropicalis* ASY2 in SWW

Mineral nutrients	Biomass yield, g L^–1^	Lipid yield, g L^–1^	Lipid content, %
With mineral nutrients	2.89 ± 0.02^a^	1.39 ± 0.02^a^	48.10 ± 0.38^a^
Without mineral nutrients	2.42 ± 0.06^b^	1.03 ± 0.02^b^	42.56 ± 0.55^b^

Values are mean (± standard error) (n = 3) and values followed by the same letter in each column are not significantly different from each other within the observation day as determined by DMRT (*p* ≤ *0.05*)

### Effect of temperature

During the growth of *C. tropicalis* ASY2 in SWW incubated at different temperatures, culture growth was not observed in the temperature range of 40–50 °C (Table 3). The maximum lipid yield (1.23 g L^–1^) with maximal amylase secretion (1.37 IU mL^–1^) and a starch consumption of 86.54% was reported when *C. tropicalis* ASY2 was grown at 30 °C. It has been reported that the maximum lipid yield of 2.12 g L^–1^ was obtained at a temperature of 38 °C when *Rhodotorula* sp. IIP-33 was grown in sugarcane bagasse hydrolysate [25].

**Table 3:** Effect of temperature on the lipid yield of *C. tropicalis* ASY2 in SWW

Temperature, °C	Biomass yield, g L^–1^	Lipid yield, g L^–1^	Lipid content, %
25	1.96 ± 0.04^c^	0.81 ± 0.02^c^	41.33 ± 0.62^c^
30	2.54 ± 0.04^a^	1.23 ± 0.02^a^	48.43 ± 0.91^a^
35	2.36 ± 0.06^b^	1.07 ± 0.02^b^	45.34 ± 1.06^b^

Values are mean (± standard error) (n = 3), and values followed by the same letter in each column are not significantly different from each other within the observation day as determined by DMRT (*p* ≤ *0.05*)

### Effect of airflow rates

**Fig. 2:**
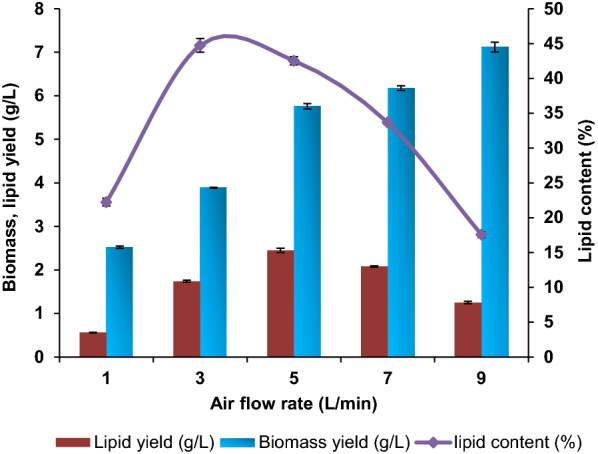
Effect of different airflow rates on the production of biomass yield, lipid yield, and lipid content of *C. tropicalis* ASY2 in ALB. The experiment was conducted in a 5 L airlift bioreactor with a working volume of 3.5 L at different airflow rates of 1 to 9 L min^–1^. Values are mean (± standard error) (n = 3)

The biomass yield, lipid yield, and lipid content on different airflow rates viz., 1, 3, 5, 7, and 9 L min^–1^ were evaluated. The highest lipid yield of 2.45 g L^–1^ was observed at an airflow rate of 5 L min^–1^ but higher lipid content (44.73%) was observed at 3 L min^–1^ (Fig. 2). After 5 L min^–1^, the lipid yield decreased gradually with an increase in the biomass yield. This is due to an increased flow rate that promotes yeast cell growth without favoring lipogenesis. The reason for an enhanced rate of cell growth was due to an increase in the rate of aeration. It may be attributed to higher oxygen supplied by an improved airflow rate [20]. Similar results were obtained by Yen H-W and Liu YX [20], who reported a higher lipid content of 53.4% at an aeration rate of 4.5 L min^–1^ but a greater lipid yield of 12.8 g L^–1^ at an aeration rate of 6 L min^–1^ in a 5 L airlift bioreactor with working volume of 3 L. It indicated that the lipid yield was essential than the lipid content of the yeast strain.

## Optimization of lipid production in an airlift bioreactor using RSM

The preliminary trial results of the culture and nutritional parameters demonstrated that the additional carbon source (starch) significantly improved the biomass and lipid yield. Also, SWW supplemented with yeast extract or peptone at 0.5% supported the growth of *C. tropicalis* ASY2. Further, the optimized culture parameters were: pH of 6 and temperature of 30 °C. The preliminary trial culture parameters were evaluated by the response surface methodology (RSM) method, with pH and temperature as the constant variables. In contrast, nutrients such as starch concentration (*A*), yeast extract (*B)*, and airflow rate (*C*) and their combination served as independent variables. The experimental CCD design matrices of the variables with varying C:N ratio (8.79, 10, 12.82, 20, 31.21, 40, and 45.45) and their response values of biomass yield, lipid yield, and lipid content of *C. tropicalis* ASY2 grown in ALB is given in Table 4.

**Table 4:** Experimental CCD design matrices of the variables with various C:N ratio and their response values of biomass yield, lipid yield, and lipid content of *C. tropicalis* ASY2 grown in ALB

Run	*A*:Starch, g L^–1^	*B*:Yeast extract, g L^–1^	*C*: Airflow rate, L min^–1^	Biomass yield, g L^–1^	Lipid yield, g L^–1^	Lipid content, %
1	20	0.5	3	3.56	1.66	46.63
2	15	0.75	5	5.56	2.42	43.53
3	20	0.5	7	6.89	2.71	39.33
4	15	0.75	5	5.52	2.23	40.4
5	15	0.75	5	5.78	2.32	40.14
6	15	0.75	5	5.46	2.25	41.21
7	6.59	0.75	5	2.21	0.35	15.84
8	10	1	3	3.04	0.79	25.99
9	15	0.75	5	4.92	2.19	44.51
10	10	1	7	6.21	1.31	21.1
11	10	0.5	3	2.94	0.83	28.23
12	20	1	3	4.18	1.56	37.32
13	15	1.17	5	6.98	2.15	30.8
14	15	0.75	5	5.34	2.42	45.32
15	20	1	7	8.25	1.65	20
16	15	0.75	8.36	8.05	1.62	20.12
17	15	0.75	1.64	2.05	0.61	29.76
18	23.41	0.75	5	5.13	2.11	41.13
19	10	0.5	7	5.12	1.45	28.32
20	15	0.33	5	5.45	2.84	52.11

### Effect of variables on the biomass yield in an airlift bioreactor

The central composite design (CCD) was run for three independent variables (starch, yeast extract, airflow rate) to maximize the lipid production in ALB. All the experimental trials were conducted according to the designed run, and each run was operated by the process conditions. The three-dimensional diagram was useful to examine the influence of each variable on the response. At each run, the maximum biomass yield (g L^–1^), lipid yield (g L^–1^), and lipid content (%) were calculated as the response values. Two-factor interactions between each variable, including *AB*, *AC*, and *BC* were also determined to evaluate the optimum response viz., biomass yield, lipid yield, and lipid content.

The quadratic model of the biomass yield gave the predicted and adjusted *R*-squared (*R*^*2*^) values of 0.96 and 0.98, respectively. The predicted *R*^*2*^ value was in good agreement with the adjusted *R*^*2*^ value since the difference between the two values was less than 0.2. Thus, the developed model could reasonably estimate the response of the system [30]. An analysis of variance (ANOVA) was conducted in this study to check the adequacy and significance of the quadratic model to explain the experimental data (see Additional file 1: Table S1 for results). The statistical significance of all model responses (biomass yield, lipid yield, lipid content, and FAME yield) was considered at a *p*-value of less than 0.05.

According to the ANOVA analysis, the *F*-value determined the significance of each term at the designed level of confidence [31]. The *F*-value of the biomass yield (94.28) implied that the model was significant at the 5% level. There was only a 0.01% chance that such a large *F*-value could occur due to noise. The *p*-value evaluated the significance of each variable and simultaneously identified the effect of each factor on the response [32, 33]. According to the *p*-values (*p* < 0.05), the linear model terms (*A*, *B,* and *C*), interactive model terms (*AC*, *BC*), and the quadratic model terms (*C*^*2*^) were significant at the 95% confidence level. Among the interactions studied, starch concentration and airflow rate resulted in a *p*-value of 0.0188 and were significant compared to other interactions. In this case, the variables *A, B*, and *C* resulted in significant model terms than the terms *AC* and *BC*.

**Fig. 3:**
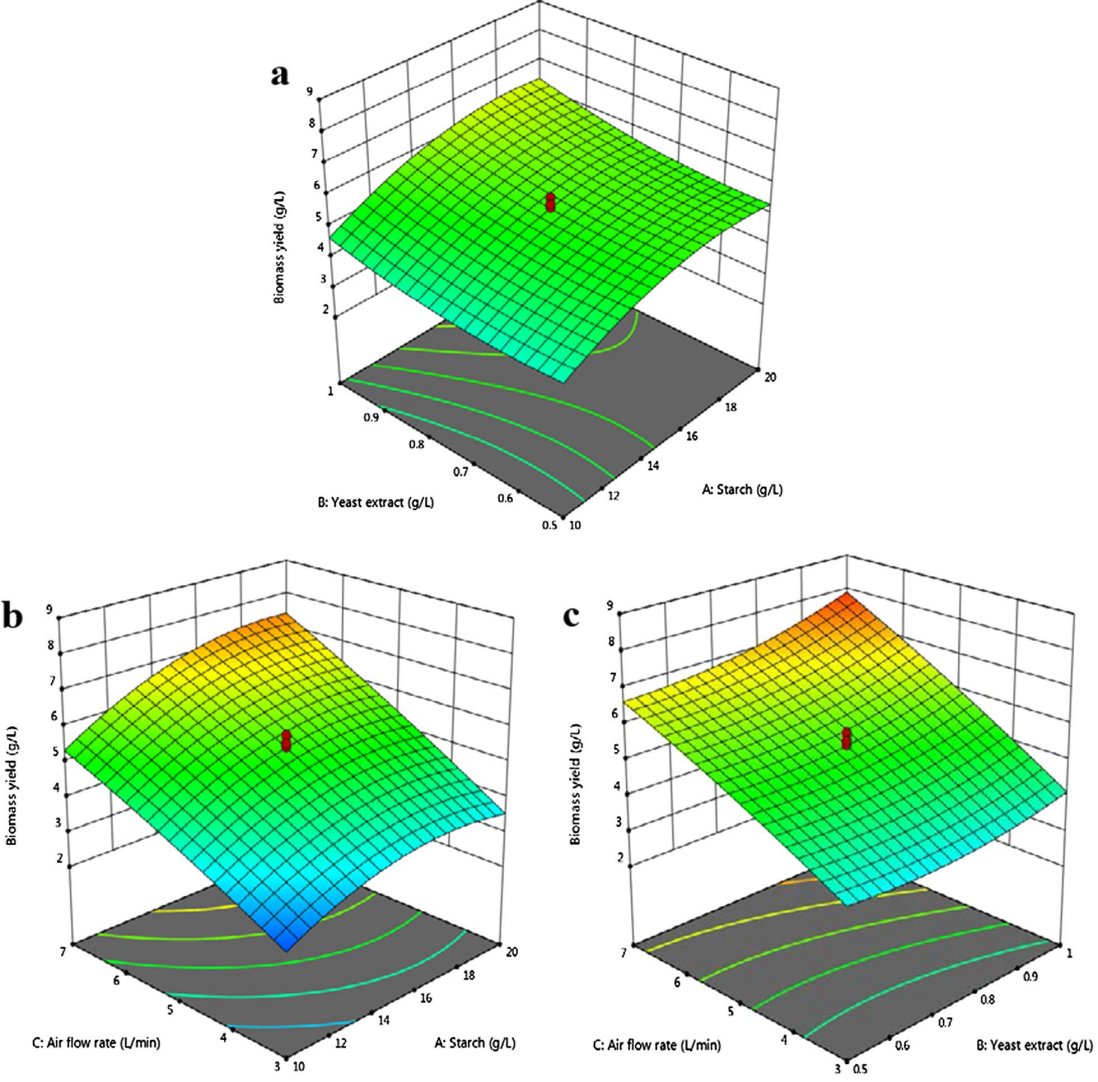
3D surface plots for the response biomass yield of *C. tropicalis* ASY2 grown in ALB. **a** Effect of starch vs. yeast extract; **b** Effect of starch vs. airflow rate; **c** Effect of yeast extract vs. airflow rate. The interactive effect is represented with the color ranging from blue to red (blue, green, red); blue is least significant, green is moderately significant, and red is highly significant

The Coefficient of Variation (CV) specified the degree of precision and was expressed as a percentage (%). The very low values of CV indicated a very high degree of precision and good reliability of the experimental values [30]. The reduction resulted in a ratio of 33.58 that indicated an adequate signal and a higher predicted response relative to the associated error. All the response surface plots were generated by maintaining one variable at its optimum level and varying the other variables within the experimental range. A three-dimensional surface expressed the interactions between the model terms. A check for the interactions was necessary to determine the significance of the model equation [33]. The interaction between yeast extract and starch was not significant. The yeast extract and airflow rate displayed significant interaction in the yield of the yeast biomass. The airflow rate and yeast extract strongly impact on the biomass yield (Fig. 3). With an increase in the aeration rate, there was a gradual increase in the biomass yield.

This interaction was consistent with the observations of Yen and Liu [20], who reported that an increase in the aeration rate provided more oxygen for the growth of the yeast biomass. The final quadratic equation for the biomass yield is given below.

$$\begin{aligned} {\text{Biomass yield }}\left( {{\text{g L}}^{{ - 1}} } \right) = & - 1.45446 + {\mkern 1mu} 0.694379A - 8.69509B + {\mkern 1mu} 0.424949C + {\mkern 1mu} 0.079AB \\ + + {\mkern 1mu} 0.025625AC + {\mkern 1mu} 0.4325BC - 0.02428A^{2} + 4.68648B^{2} - 0.02975C^{2} \\ \end{aligned}$$

### Effect of variables on the lipid yield in an airlift bioreactor

The predicted and adjusted *R*-squared (*R*^*2*^) values for the lipid yield of the quadratic model were 0.85 and 0.96, respectively. The predicted *R*^*2*^ value was in agreement with the adjusted *R*^*2*^ value. For lipid yield response, analysis of variance (ANOVA) was performed to interpret the effects and significance of the interaction of variables (see Additional file 1: Table S2 for results). The *F*-value of 48.75 implied that the model was significant. Among the interactions, *AB* and *BC* resulted in *p*-values of 0.0399 and 0.0287, respectively. In general, a *p*-value of less than 0.05 (*p* < *0.05*) indicates the significance of the model term. According to the *p*-values (*p* < *0.05*), the linear model terms (*A*, *B,* and *C*), quadratic model terms (*A*^*2*^, *C*^*2*^), and the interactive model terms (*AB*, *BC*) were all significant at 95% confidence level. Therefore, the model was adequate.

**Fig. 4:**
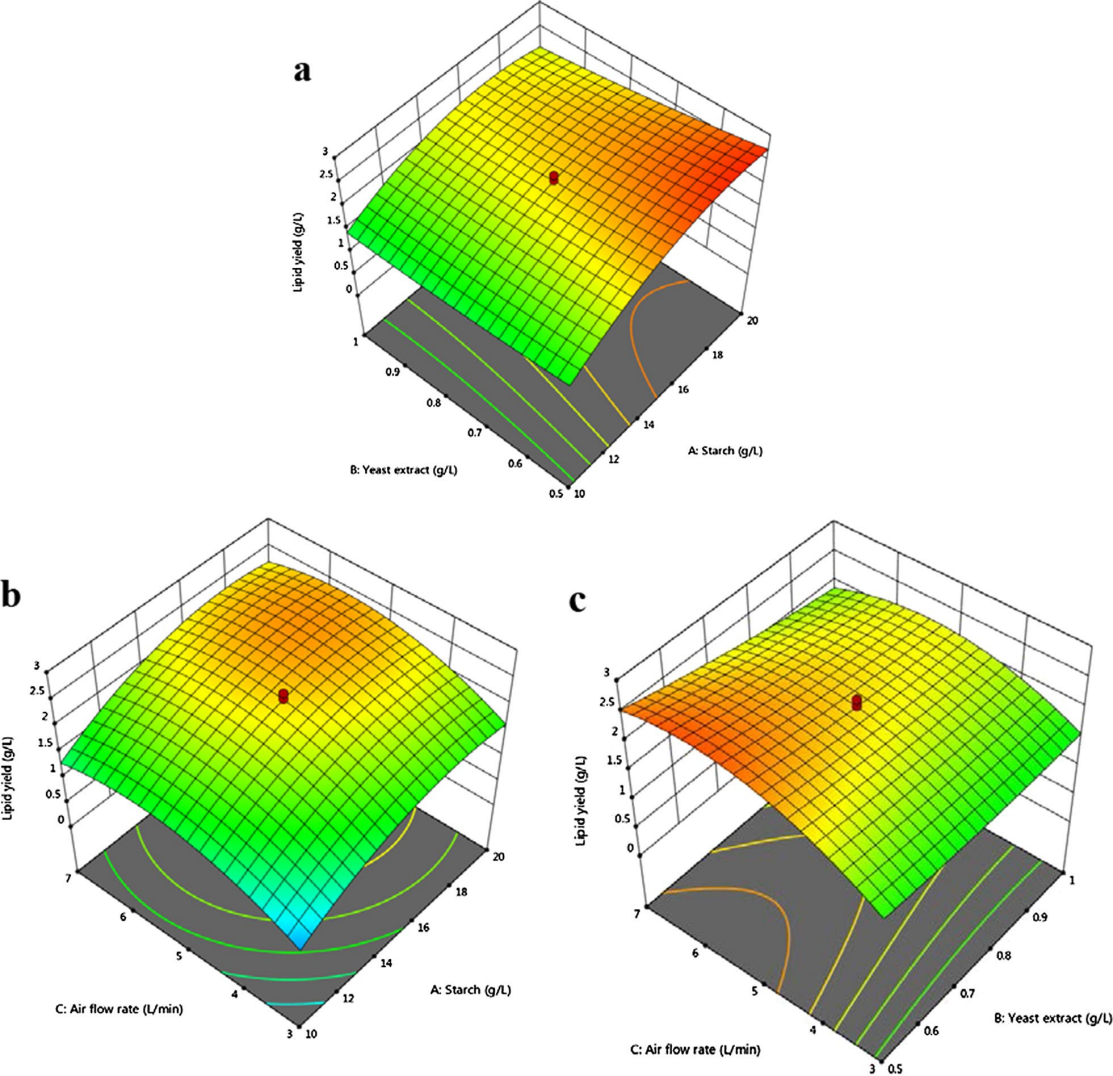
3D surface plots for the response lipid yield of *C. tropicalis* ASY2 grown in ALB. **a** Effect of starch vs. yeast extract; **b** Effect of starch vs. airflow rate; **c** Effect of yeast extract vs. airflow rate. The interactive effect is represented with the color ranging from blue to red (blue, green, red); blue is least significant, green is moderately significant, and red is highly significant

The lipid yield response resulted in a CV value of 8.28%, indicating that the model exhibited good precision and experimental reliability. In this case, the adequate precision for lipid yield resulted in a ratio of 22.46 that indicated an adequate signal. The response surface plots for the lipid yield are shown in Fig. 4.

$$\begin{aligned} {\text{Lipid yield}}({\text{g L}}^{{{-}{1}}} ) = & - {7}.{15744 } + \, 0.{639135}A + \, 0.{837251}B + { 1}.{43653}C - 0.0{98}AB - {1}.{\text{36E}} - {16}AC \\ - 0.{265}BC - 0.0{1585}A^{2} + \, 0.{816925}B^{2} - 0.{1}0{921}C^{2} \\ \end{aligned}$$

### Effect of variables on the lipid content in an airlift bioreactor

**Fig. 5:**
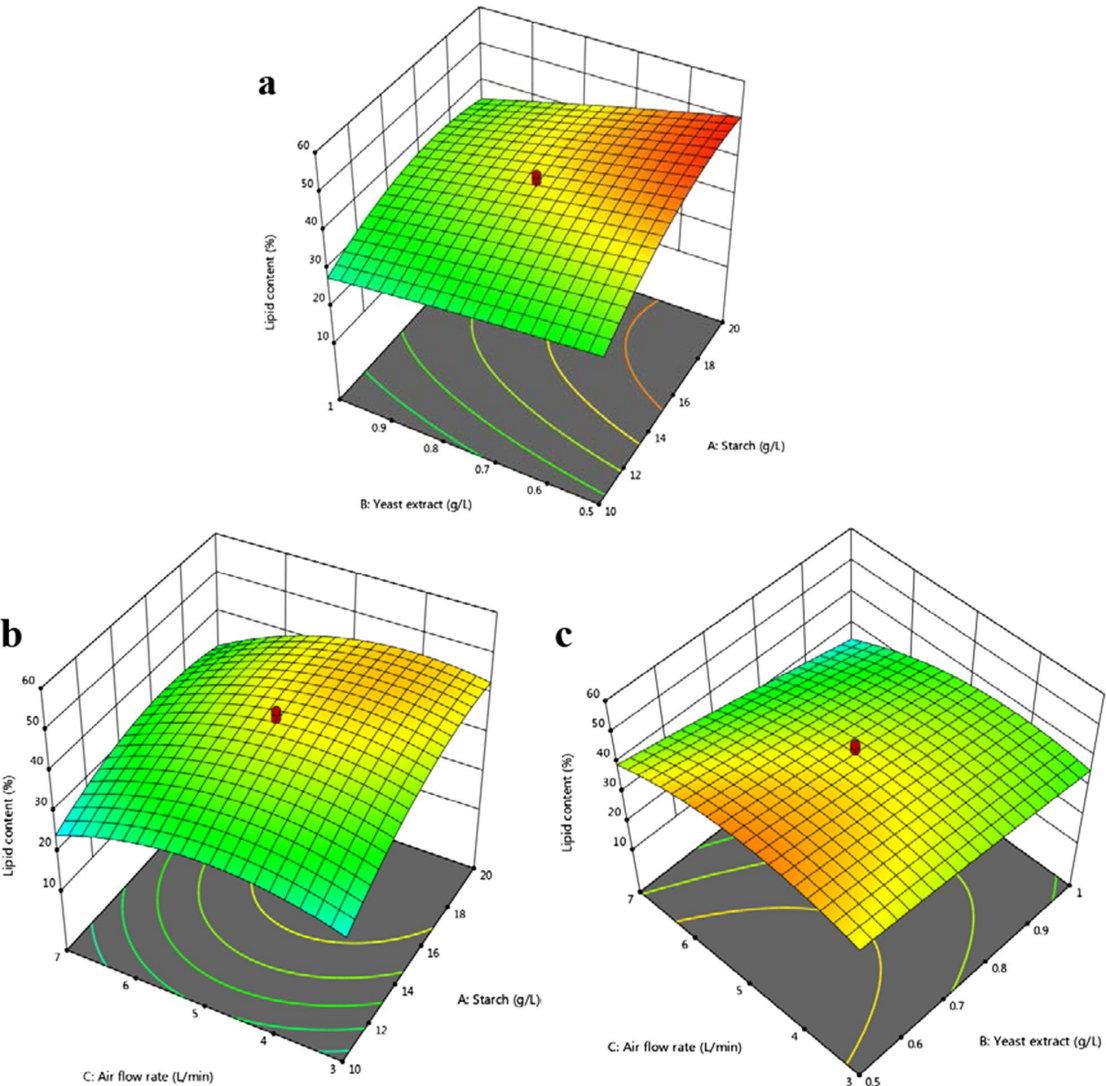
3D surface plots for the response lipid content of *C. tropicalis* ASY2 grown in ALB. **a** Effect of starch vs. yeast extract; **b** Effect of starch vs. airflow rate; **c** Effect of yeast extract vs. airflow rate. The interactive effect is represented with the color ranging from blue to red (blue, green, red); blue is least significant, green is moderately significant, and red is highly significant

The predicted and adjusted *R*-squared (*R*^*2*^) values for the lipid content of the quadratic model were 0.85 and 0.95, respectively. The predicted *R*^*2*^ value was in reasonable agreement with the adjusted *R*^*2*^ value (see Additional file 1: Table S3 for results). The *F*-value of 37.25 implied that the model was significant. According to the *p*-values (*p* < 0.05), the linear model terms (*A*, *B*, and *C*), quadratic model terms (*A*^*2*^, *C*^*2*^), and the interactive model terms (*AB*, *AC*) were significant at 95% confidence level. The interactions between *AB* and *AC* resulted in *p*-values of 0.0199 and 0.017, respectively. In this case, the variables *A* and *B* resulted in more significant model terms than *AB* and *AC*. The lipid content response exhibited a CV value of 7.09% that indicated a model with better experimental reliability. In the present case, the adequate precision resulted in a ratio of 18.58 that was an adequate signal. The response surface plots for response lipid content are shown in Fig. 5.

The interaction between the starch concentration and yeast extract demonstrated an increase in the lipid content from 40 to 50% when the starch content increased from 12 to 20 g L^–1^. Among the interactions studied, starch concentration played a major role in determining the lipid content. A maximum of 45.96% lipid content was observed at a airflow rate of 6 L min^–1^. Yen H-W, Chang J-T and Chang J-S [34] observed a lipid content of 34.3% at an aeration rate of 6 L min^–1^ when *R. glutinis* was cultured in ALB using rice straw hydrolysate and crude glycerol as the substrate. An lipid content of 47.9% was observed in the batch fermentation of ALB when marine-derived oleaginous yeast *R. glutinis* TJY15a was cultured using cassava starch hydrolysate [35]. The following equation expressed an empirical relation between the independent and dependent variables.

$$\begin{aligned} {\text{Lipid content }}\left( \% \right) = & - {92}.{5519 } + { 9}.{86}00{5}A + { 35}.{39785}B + { 2}0.{44854}C - {1}.{918}AB \\ - 0.{24775}AC - {3}.{75}BC - 0.{19931}A^{2} - {6}.{356}0{6}B^{2} - {1}.{559}0{5}C^{2} \\ \end{aligned}$$

In view of economic viability, peptone and other cultivation amendments were considered in preliminary trials. For example, at the lab-scale level, the amount of yeast extract used per batch (3.5 L) of ALB at optimal conditions was 1.75 g, which costs around 1.68 INR. All the amendments used in the study were only at sub-optimal level. Given the economic application of this technology for large-scale commercial use, yeast extract and peptone may be substituted with more cost-effective agro-waste by-products such as extruded bean [36], rice bran extracts [37], and dried spent yeast [38].

## FAME profile of *C. tropicalis* ASY2 grown in airlift bioreactor

**Fig. 6:**
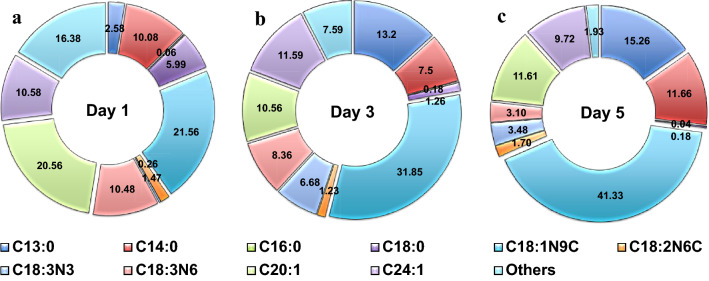
FAME profile composition (%) of *C. tropicalis* ASY2 cultivated in ALB (**a**) Day 1 (**b**) Day 3 (**c**) Day 5. Note: C13:0 tridecylic acid, C14:0 myristic acid, C16:0 palmitic acid, C18:0 stearic acid, C18:1N9C oleic acid, C18:2N6C linoleic acid, C18:3N3 alpha-linoleic acid, C18:3N6 gamma-linoleic acid, C20:1 eicosenoic acid, C24:1 nervonic acid

In the present investigation, the yeast strain produced different chain lengths of fatty acids ranging from C4 to C24 during different incubation days. Besides the chain length, the composition and concentration of fatty acids also varied. It produced almost all the primary and other fatty acids (tridecylic acid, myristic acid, palmitic acid, stearic acid, oleic acid, linoleic acid, alpha-linoleic acid, gamma-linoleic acid, eicosenoic acid and nervonic acid), which are found in vegetable oils [39, 40]. Oleic, stearic, linoleic, and linolenic acids are important fatty acids for biodiesel generation. A higher content of oleic acid alone was found on 5th day (41.33%) compared to 3rd (31.85%) and 1st (21.56%) day (Fig. 6). In comparison to jatropha (39.08%) and soybean oils (31.14%), the FAME profile had the highest concentration of oleic acid (41.33%) [41]. The differences in FAME proportions are noted, which could be related to nutrient or C:N ratio variation/depletion in the SWW [17] and growth conditions that affect yeast growth [42].

## Direct transesterification of yeast biomass into biodiesel

In a direct or in situ transesterification, dry biomass lipids are converted into FAMEs in a single step. The procedures for lipid extraction and conversion to FAME are combined to render the entire process less tedious and energy-efficient. This kind of reduction of steps guarantees a reduced loss of the materials. In the direct transesterification process, the catalyst plays a major role in the efficient conversion of FAME. Lotero et al. [43] reported that the selection of the catalyst is dependent on the feedstock to be used in the transesterification process.

Lotero et al. [43] also stated that, base-catalyzed processes need a feedstock that contains free fatty acid (FFA) that does not exceed 0.5% of the overall lipid weight; anhydrous glycerides and alcohol are needed to prevent saponification. These reactions are generally faster and typically occur at lower temperatures than acid-catalyzed processes. However, they cannot be used for high FFA containing feedstocks [44, 45]. In base-catalyzed transesterification reactions, feedstocks with higher FFA content (> 2.5%, w/w) preferred saponification reactions instead of FAME conversion at higher temperatures, until any pretreatment was used [46–48]. In the present study, the moisture content of the yeast biomass was 7.21% and the free fatty acid content of yeast oil was 2.54%. This value suggested that acid-catalyzed transesterification was ideal for carrying out further experiments to prevent saponification reactions.

### Effect of the influential factors for maximum FAME yield

#### Effect of the catalyst concentration

**Fig. 7:**
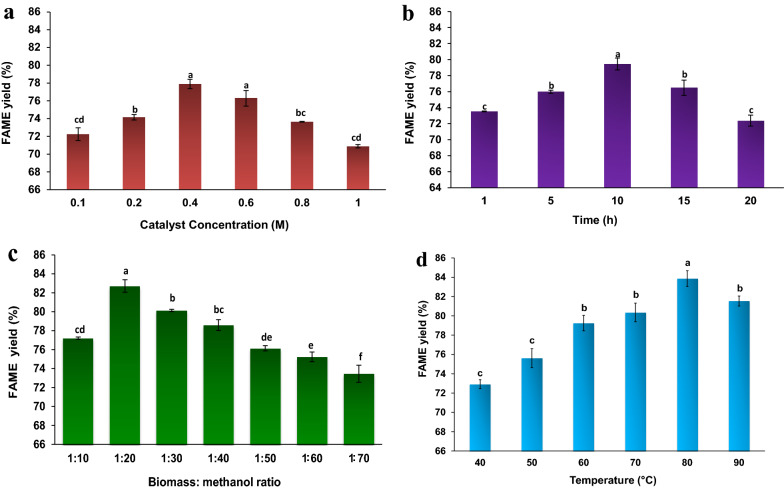
Study of influential parameters on FAME yield in transesterification process. **a** Effect of different catalyst concentrations; **b** Effect of different time; **c** Effect of various biomass: methanol ratio; **d** Effect of different temperature. The experiment was conducted at a shaking speed of 150 rpm using one gram of lipid-accumulated yeast biomass at different catalyst concentrations (0.1 to 1 M), biomass: methanol ratios (1:10 to 1:70), times (1 to 20 h), and temperatures (40 to 90 °C). Values are mean (± standard error) (n = 3) and same alphabets in each column are not significantly different from each other within the observation day as determined by DMRT (*p* ≤ *0.05*)

In this study, six different concentrations of acid (H_2_SO_4_) were evaluated for their catalytic action. Figure 7a shows the FAME yields for different catalyst concentrations. The FAME yield increased upon increasing the catalyst concentration from 0.1 to 0.4 M. The maximum FAME yield of 77.89% was observed at an acid catalyst concentration of 0.4 M. However, the yield dropped slightly as the catalyst quantity increased further. The acid catalyst further destabilized the cell membrane in the yeast and allowed the organic solvent to enter inside the intracellular lipid layer. The ideal use of acid as the catalyst is industrially and technically important because excessive use of acid is not commercially beneficial. Moreover, surplus acid in the reaction mixture provokes side reactions that can lead to the polymerization of unsaturated fatty acids [49].

### Effect of the reaction time

To study the FAME yield, the reaction times were varied from 1 to 20 h with 0.4 M catalyst concentration and biomass: methanol ratio of 1:20 (w/v) at 80 °C. During the initial hours, the FAME yields were very low and showed a maximum at 10 h. After this time, the yield declined, likely due to the FAME degradation because of the reversible nature of the transesterification reaction (Fig. 7b). A FAME yield of 79.45% was obtained at 10 h of acid-catalyzed reaction; this yield was 7 h faster than that reported by Thliveros P, Kiran EU and Webb C [50].

### Effect of the biomass: methanol ratio

The effect of methanol loading on transesterification process is fundamental from an industrial point of view because recycling the solvent is a costly affair. Therefore, to achieve maximum conversion of FAME, an ideal amount of methanol should be used in the process (Fig. 7c). The FAME yield increased by increasing the methanol concentration up to 1:20 (w/v), after that subsequently decreased. Enhancing the concentration of methanol improved the polarity of the mixture, which induced the reaction toward product formation. Thus, increasing the amount of methanol beyond the safe level dilutes the biomass and leads to decreased efficiency of conversion. Therefore, a maximum FAME yield of 82.72% was attained with the biomass: methanol ratio of 1:20 (w/v) (Fig. 7c). This result was substantiated by Thliveros P, Kiran EU and Webb C [50], who achieved a higher FAME yield of 77.9% during the biomass: methanol ratio optimization for *R. toruloides*.

### Influence of the reaction temperature

The FAME yield was found to increase when the temperature rose from 40 to 90 °C. A maximum yield of 83.86% was obtained at a temperature of 80 °C and biomass: methanol ratio of 1:20 (w/v) (Fig. 7d). Increasing the temperature of the process reduces the viscosity of the lipids with faster reaction speeds and slower times but only to a critical level; above this level, the FAME yield begins to decrease [50, 51]. Eevera et al. [52] and Leung and Guo [47] also observed a decrease in the biodiesel yield when the reaction temperature rose above the optimum level. An increased reaction temperature accelerates the triglyceride saponification process and simultaneously consumes the catalyst. Thus, the reaction temperature should be retained at the optimal level to achieve the maximum yield of FAME.

## Optimization of the transesterification process via RSM

In this process optimization, the CCD design in RSM was adopted to assess the parametric effect of three variables on the response FAME yield (%). The variables were methanol (*A*), catalyst concentration (*B*), and time (*C*). Their response FAME yield is shown in Table 5. The predicted and adjusted *R-*squared (*R*^*2*^) values for the FAME yield of the quadratic model were 0.70 and 0.89, respectively. The predicted *R*^*2*^ value was in agreement with the adjusted *R*^*2*^ value since the difference between two values was less than 0.2.

**Table 5:** Experimental CCD design matrices of their variables and their response values of FAME yield in direct transesterification process

Run	*A*:Methanol, mL	*B*:Catalyst conc., M	*C*:Time, h	FAME yield, %
1	30	0.3	7.5	80.58
2	30	0.3	3.30	69.89
3	13.18	0.3	7.5	78.96
4	40	0.2	5	66.52
5	30	0.3	7.5	77.74
6	20	0.4	5	86.56
7	40	0.4	10	72.34
8	40	0.2	10	68.54
9	30	0.3	7.5	79.53
10	30	0.3	7.5	81.97
11	20	0.4	10	83.65
12	40	0.4	5	69.72
13	46.82	0.3	7.5	61.58
14	30	0.47	7.5	84.56
15	30	0.3	7.5	81.07
16	20	0.2	10	76.12
17	30	0.13	7.5	75.82
18	20	0.2	5	82.71
19	30	0.3	11.70	71.56
20	30	0.3	7.5	77.14

**Fig. 8:**
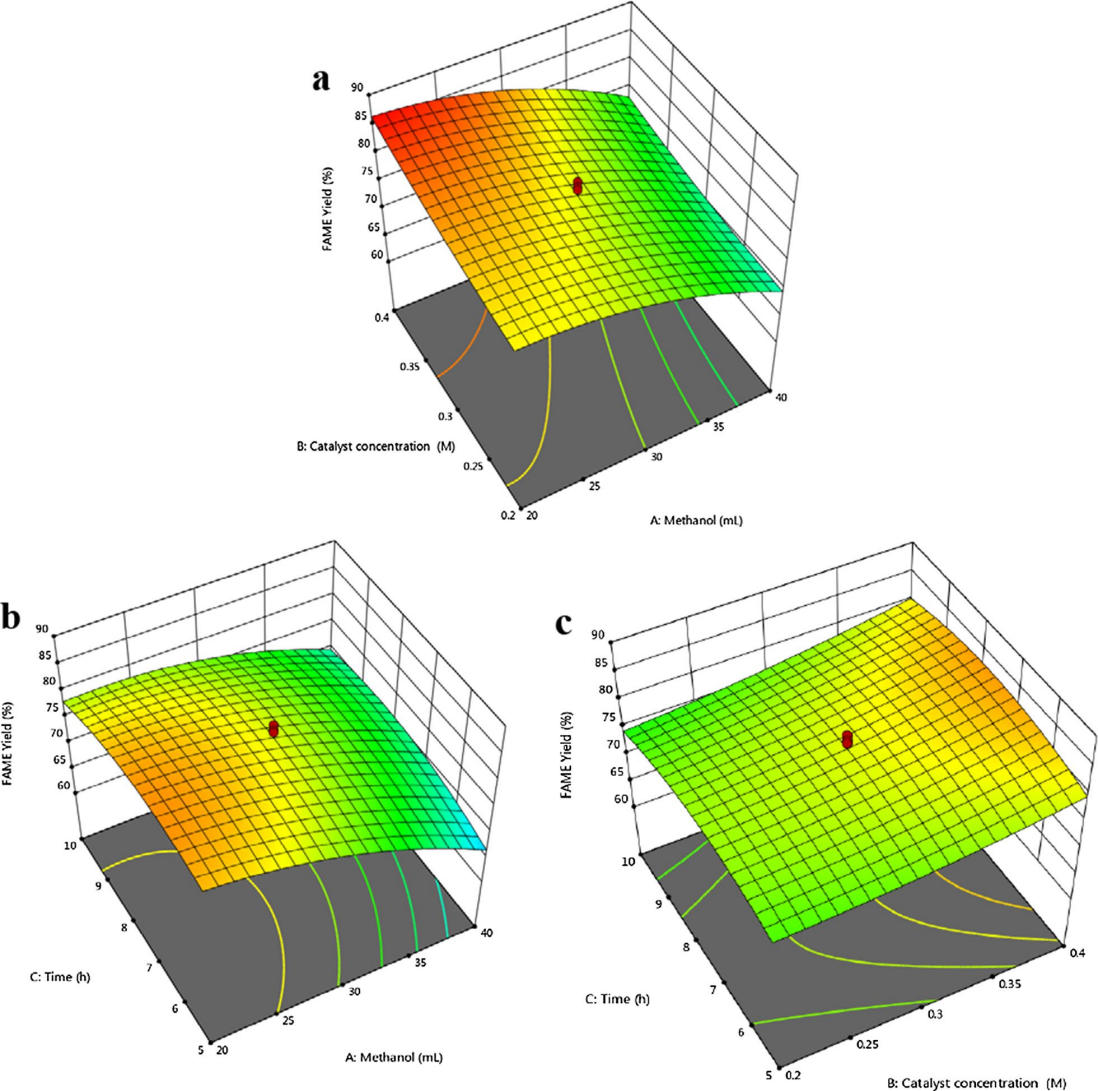
3D surface plots for the response FAME yield of *C. tropicalis* ASY2 yeast biomass in transesterification process. **a** Effect of methanol vs. catalyst concentration; **b** Effect of methanol vs. time; **c** Effect of catalyst concentration vs. time. The interactive effect is represented with the color ranging from blue to red (blue, green, red); blue is least significant, green is moderately significant, and red is highly significant

The ANOVA results for the FAME yield response (Additional file 1: Table S4) indicated an *F*-value of 18.75, implying that the model was significant. According to the *p*-values (*p* < *0.05*), the linear model terms (*A* and *B*), quadratic model terms (*A*^*2*^, *C*^*2*^), and the interactive model terms (*AC*) were significant at 95% confidence level; thus, the model was adequate. The CV value of 2.87% indicated that the model exhibited good precision and experimental reliability. The adequate precision ratio of 15.49 indicated an adequate signal. The response surface plots for the response FAME yield are shown in Fig. 8. The interaction between methanol and the catalyst concentration illustrated an increase in the FAME yield from 80 to 85% when the catalyst concentration increased from 0.2 to 0.4 g L^–1^. Simultaneously, the yield decreased from 80 to 75% when the biomass: methanol ratio reached 1:20 (w/v). An excess methanol loading could have diluted the concentration of the biomass and reduced the conversion of oil, and consequently, decreased the FAME yield. These results appeared to be consistent with the results of Alamu OJ, Waheed MA and Jekayinfa SO [53], who noted an increase in the yield of biodiesel when the ratio of methanol to oil increased only up to a threshold level. The interaction between the catalyst concentrations and reaction times revealed that the yield increased up to a certain level of time and then decreased. This could be due to FAME degradation and an increasing saponification degree [52, 54]. The interaction between the methanol concentration (*A*) and time (*C*) was significant (*p* < 0.05). The quadratic second-order polynomial equation of FAME yield is shown below.

$$\begin{aligned} {\text{FAME yield}}, \, \% =\, & {63}.{35155 } + \, 0.{73}00{39}A - {16}.{8143}B + { 3}.{55181}C - 0.{5475}AB + \, 0.0{7}0{7}AC \\ + { 2}.{14}BC - 0.0{2817}A^{2} + { 69}.0{1785}B^{2} - 0.{42499}C^{2} \\ \end{aligned}$$

## Energy consumption of conventional and in situ transesterification process

The conventional transesterification step consists of biomass drying, cell disruption, lipid extraction, and transesterification. For biomass drying in a hot air oven (600 W, 12 h), the energy consumption was about 7.2 kWh. The energy requirement for cell disruption in the sonicator was 61.2 kWh kg^–1^ of biomass. The lipid extraction was carried out in a shaker that consumed around 140 kWh kg^–1^ of biomass. The hot plate magnetic stirrer used for the transesterification step consumed about 4 kWh kg^–1^ of biomass. Hence, bypassing the lipid extraction step with a direct transesterification step reduced the energy consumption by around 64.57% (Table 6).

**Table 6:** Comparative analysis of the total energy consumption and cost involved in both conventional and in situ transesterification processes

Technique and its unit involved	Conventional	In situ
Biomass drying (Hot air oven)	7.2	7.2
Cell disruption (Sonicator)	61.2	61.2
Lipid extraction (Orbital shaker)	140	–
Transesterification (Hot plate magnetic stirrer)	4	6.85
Total energy consumption (kW h)	212.4	75.25
Energy saved, %	64.57	
Cost of electricity per unit (on average)	5	5
Total cost to process 1 kg biomass (INR)	1062	376.25
Cost saved per kg of biomass (INR)	685.75	
Production cost per batch of ALB (INR)	295.11	

Energy consumption per unit operation/process (kWh kg^–1^ biomass)

Further, the energy cost calculated for the conventional and in situ processes accounted for about 1062 INR and 376.25 INR (calculated at the rate of 5 INR per kWh), respectively, saving 685.75 INR for processing 1 kg biomass. Thus, the in situ method can be extended to the pilot or industrial scale as it was more sustainable and feasible in terms of solvent use, energy efficiency, time consumption, and reduced capital costs.

## Conclusion

The use of oleaginous yeasts for lipid production is of great interest since lipids can accumulate up to 70 percent of their biomass. The present work resulted in maximized lipid yield, and consequently, biodiesel was derived from the oleaginous yeast, *Candida tropicalis* ASY2. Under optimized conditions in the bioreactor, the highest biomass yield of 5.992 g L^–1^, lipid yield of 2.68 g L^–1^, and lipid content of 45.96% were obtained by using 15.33 g L^–1^ of starch content and 0.5 g L^–1^ of yeast extract at the airflow rate of 5.992 L min^–1^. The direct transesterification process yielded a higher FAME yield of 86.56% at the optimized conditions of 1:20 biomass: methanol ratio, 0.4 M of the catalyst concentration, and 6.85 h of time. In the in situ method, we could save around 64.57% of energy and a cost of 685.75 INR for processing 1 kg of yeast biomass. Thus, the biodiesel produced from the SWW using oleaginous yeast can be a potential source for the development of a sustainable environment in the near future.

## Methods

### Materials

Sago factory wastewater samples were collected from Sri Selliamman Sago Industries, Alavaipatty, Rasipuram Taluk, Namakkal District of Tamil Nadu, India. The samples were collected under aseptic conditions using a grab sampler in a clean, sterile plastic container to avoid the environmental/biological contaminants and stored at 4 °C until further use. The physicochemical properties of SWW used in the present experiments were characterized in our earlier study [17, 19].

### Chemicals and reagents

All materials for culture medium (yeast extract, peptone, malt extract, glucose, starch) and other chemicals, such as methanol, sulfuric acid, chloroform, picric acid, sodium carbonate, and sodium sulfate, were bought from HiMedia Laboratories (Mumbai, India). FAME standards were purchased from Sigma-Aldrich (St Louis, MO, USA). The 5-L capacity airlift bioreactor was designed and manufactured at M/S Science World, Coimbatore, India.

### Microorganism and culture conditions

*C. tropicalis* ASY2, oleaginous yeast (GenBank Accession number MH011502), was isolated from SWW by the biotrap enrichment method [17]. Optimally grown *C. tropicalis* ASY2 (30 °C, pH 6) was used in this experimental study. The stock culture was maintained on the YEME medium (composition in g L^–1^: yeast extract, 3.0; malt extract, 3.0; glucose 10.0; peptone 5.0; pH 6.0).

### Cultivation conditions of yeast strain

The collected raw SWW samples were used as such without employing any pretreatment in the present study (e.g., filtration and acid) except for pH and starch level adjustment. The pH of raw SWW was adjusted to 6 from 4.67, and its starch content was made to 10 g L^–1^ from 4.82 g L^–1^ [17, 24]. The SWW samples were autoclaved prior to use in various assays to avoid growth of contaminating microbes. The yeast strain *C. tropicalis* ASY2 was inoculated at a density of 10^6^ cells mL^−1^ in a 250 mL Erlenmeyer flask containing 50 mL of sterile SWW (C:N ratio of 10:1) was used for cultivation of yeast for five days at 30 °C in an incubator shaker (150 rpm) without any additional nutrients since raw SWW itself contains nitrogen in the form of ammoniacal (5.48 mg L^–1^) and nitrate-nitrogen (3.10 mg L^–1^) [17]. All experiments were carried out under aseptic conditions and only sterile SWW was used as a growth medium for inoculation of pure culture of *C. tropicalis* ASY2.

### Determination of the biomass yield, lipid yield, and lipid content

Periodically, the SWW samples, along with the yeast biomass, were withdrawn and analyzed for lipid production. The dry cell weight of the yeast (g L^–1^) was gravimetrically determined as follows: An aliquot of 50 mL of the culture sample was centrifuged at 6000 rpm (10 min) and the biomass pellet was washed twice with sterile distilled water and dried in a hot air oven at 40 °C until a constant weight (usually 24 h) was achieved. The lipid yield (g L^–1^) and lipid content (%) were determined according to the procedures followed by Folch J, Lees M and Stanley GHS [55]. The residual starch and residual glucose (amylase activity) were determined as described earlier [17].

### Examination of influential factors for higher lipid production of *C. tropicalis* ASY2

Influential parameters, such as different carbon sources (arabinose, glucose, galactose, fructose, xylose, mannose, and starch—each at a fixed concentration of 0.5%), starch concentrations (10 to 80 g L^–1^), nitrogen sources (urea, peptone, tryptone, yeast extract, ammonium chloride, and ammonium sulfate—each 0.5% concentration), pH (5 to 7), mineral salts, and temperature were optimized for improving the lipid yield of strain ASY2 in SWW, cultivated for five days.

### System descriptions and operational conditions

The airlift bioreactor (ALB) of 5 L capacity consisted of the riser, downcomer, gas separator, aerator, air rotameter (for regulating the airflow rate), air sparger, non-return valve, Teflon stopcock (for controlling the airflow rate), and other accessories. The volume of wastewater used for each run is 3.5 L. The aerator outlet was connected to the input of the air rotameter. The air rotameter output was connected to the air sparger inlet. To make a closed-loop circulation, the air from the aerator was supplied to the air sparger with the help of the air rotameter. The purpose of the Teflon stopcock was to regulate the flow rate of the working liquid. A non-return valve was mounted before the air sparger to prevent the backflow of fluid into the air inlet line at low airflow rates.

**Fig. 9:**
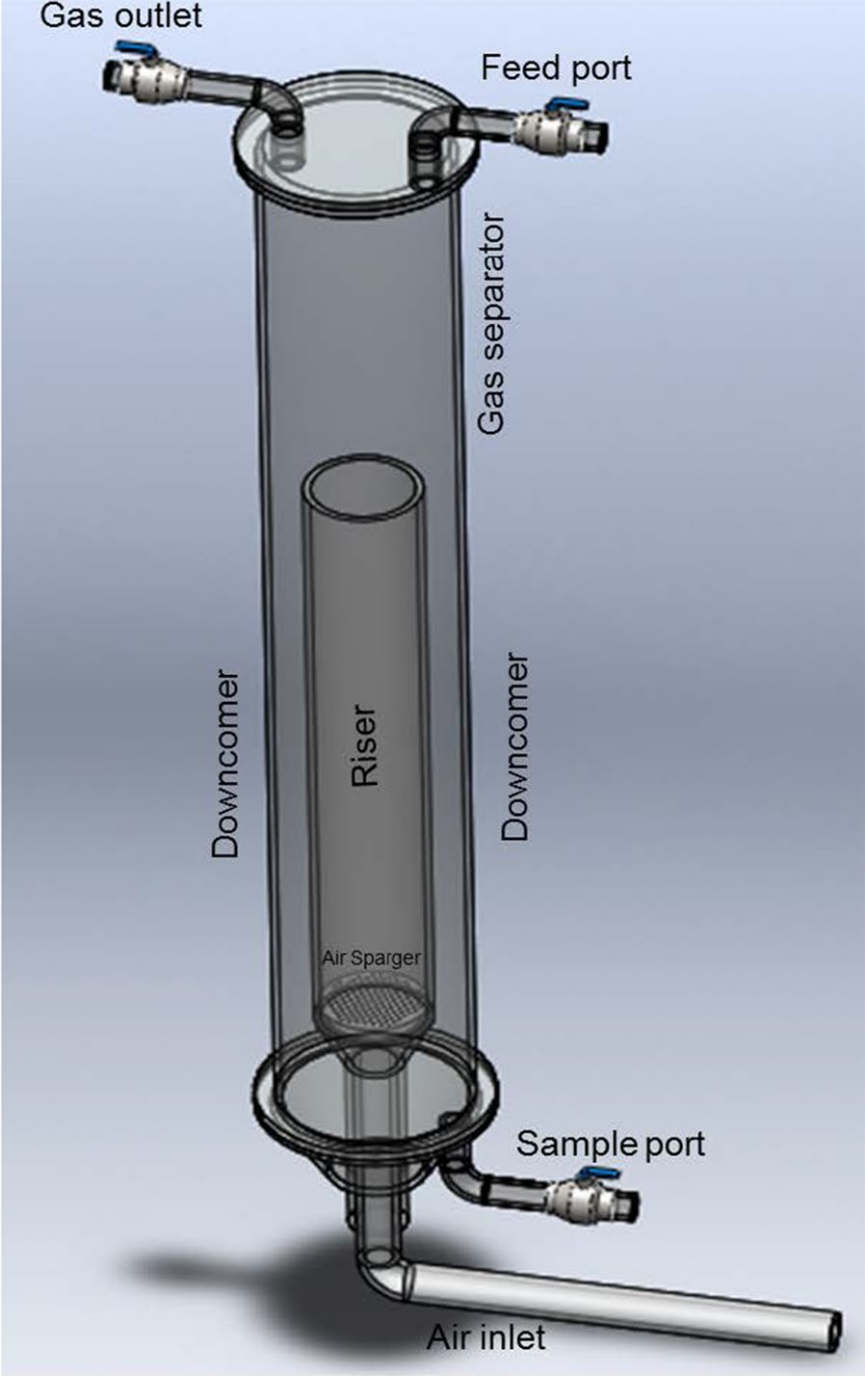
Schematic diagram of 5 L capacity airlift bioreactor (ALB)

The pH, temperature, and DO (dissolved oxygen) probes were used to measure the pH changes, temperature, and DO content, respectively. Feed port and gas output ports were located at the top of the bioreactor to inoculate the oleaginous yeast culture in the SWW and gas release, respectively. The sample port was located at the bottom of the bioreactor for sample collection at regular time intervals. The material used for bioreactor fabrication was borosilicate glass. It has very low CTE (Coefficient of Thermal Expansion), which is greatly resistant to thermal shock, durable, and resistant to various biochemical changes. The schematic diagram of 5 L capacity airlift bioreactor is given in Fig. 9.

### Experimental design

Response Surface Methodology (RSM) is a set of mathematical and statistical techniques used to create, enhance, and optimize processes and also determine the relative importance of several process parameters even in the presence of complex reaction conditions. The main objective of RSM is to optimize the process variables. CCD (Central Composite Design) and Box-Behnken designs were the principal response surface methodologies used in experimental design. RSM was used to optimize the operating conditions in 5 L airlift bioreactors to attain higher lipid productivity using CCD. The response was assumed to be affected by three independent variables: the carbon source (*A*), nitrogen source (*B*), and airflow rate (*C*). The levels of the independent variables were fixed based on the preliminary trial. The following responses were estimated in the optimal study: biomass yield (g L^–1^), lipid yield (g L^–1^), and lipid content (%). The range of the three selected independent variables used for lipid production is furnished below.

- Carbon source (Starch) = 10–20 g L^–1^
- Nitrogen source (Yeast extract) = 0.5–1 g L^–1^
- Airflow rate = 3–7 L min^–1^

To obtain higher lipid production in ALB, the pH adjusted SWW was sterilized with appropriate quantities of starch and yeast extract. Each experimental run in ALB was carried out for five days (30 °C, pH: 6) continuously with a working volume of 3.5 L under batch mode. The inoculum of *C. tropicalis* ASY2 was inoculated at a density of 10^6^ CFU mL^–1^ through the feed port in the bioreactor containing sterile SWW. The closed-loop circulation in ALB enables thorough mixing of SWW and inoculum, which accelerated the oxygen supply to the yeast strain ASY2 and promoted the growth of yeast and subsequent lipid production in ALB. After five days of experimental run, yeast cell biomass was separated by gravity settling and sonicated. Its cell count was measured by flow cytometry (BD Biosciences, USA). Then, the wet yeast biomass was dried in a hot air oven at 40 °C until the constant weight reached [18, 51]. The same procedure was followed to reproduce the experimental run.

### Moisture content estimation

In the direct transesterification process, the moisture content in the yeast biomass affects the overall biodiesel yield [56, 57]. Hence, the moisture content of yeast biomass was determined according to the ASTM E871 method using a hot air oven at a temperature of 105 °C for 24 h [18].

### Direct transesterification of yeast biomass and analyses of FAME

The traditional method of converting yeast SCO to biodiesel includes the following steps in this order: cell destruction, oil extraction, separation, and transesterification. To reduce energy consumption and the number of solvents used in these steps, the above steps were combined into one usually described as direct transesterification [58]. 1 g of dry biomass in different volumes of methanol (biomass to methanol ratio) was added to a 250 mL conical flask and kept on a hot plate magnetic stirrer. H_2_SO_4_ at different concentrations was dissolved in methanol and used as the catalyst. The mixture was heated at 80 °C from 1 to 20 h with vigorous mixing and cooled to ambient temperature. 1 mL of distilled water is added and followed by centrifugation at 1500 rpm for 5 min. The water residue from the obtained FAME was removed by adding anhydrous Na_2_SO_4_ [59].

The extracted FAME esters from lower phase were analyzed by a gas chromatography instrument (Perkin Elmer Clarus 680 GC, USA) equipped with a flame ionization detector (FID) and an Elite-5 Capillary Column (30 m length $\times$ 0.25 mm I.D.$\times$ 0.25 µm film thickness). The injection temperature was 220 °C; the initial column temperature was 160 °C, and the final temperature of 190 °C was achieved by increasing the temperature at the rate of 3 °C per min; the detector temperature was 270 °C. The carrier gas helium was used at a flow rate of 1.3 mL min^–1^. The FAME composition of lipids was determined by comparing the retention times and peak areas of the samples with the standard FAME mixtures (Sigma).

### Examination of influential factors for higher FAME yield in direct transesterification process

Influential parameters of transesterification process were biomass: methanol ratio (1:10 to 1:70), H_2_SO_4_ concentration (0.1 to 1 M), and reaction time (1 to 20 h) were optimized for improving the FAME yield of strain ASY2 cultured in the ALB for five days.

### Optimization of direct transesterification process

RSM was employed to optimize the operating conditions of direct transesterification process of the yeast biomass to attain a higher biodiesel yield. The response was expected to be influenced by three independent variables: biomass: methanol ratio (*A*), catalyst concentration (*B*), and time (*C*). The levels of independent variables were fixed based on the above mentioned preliminary trial. All the experimental runs were carried out at a temperature of 80 °C with varying biomass: methanol ratio, catalyst concentration and time. The range of the selected independent variables used for the transesterification process is furnished below.

- Biomass: methanol ratio = 1:20–1:40
- Catalyst concentration = 0.2–0.4 M (H_2_SO_4_)
- Time = 5–10 h

### Energy consumption in conventional and in situ transesterification

The energy consumption was calculated at each level of the transesterification process based on the following equation [51]:

1 $${\text{Energy consumption by unit operation}}/{\text{process }}\left( {\text{kW h}} \right) = \,{\text{Power rating of the instrument }}\left( {{\text{kW}}} \right)\times {\text{Time of operation }}\left( {\text{h}} \right)$$

2 $$\% {\text{Energy\,saved}}=\frac{\text{Energy\, consumed\, in\, (Conventional \,method}-{\text{In situ\, method)}}}{\text{Energy \,consumed\, in \,conventional \,method}} \times 100$$

## Supplementary Information

**Additional file 1.** Additional tables.

## Data Availability

All data generated or analyzed during this study are included in this published article.

## Abbreviations

ALB: Airlift bioreactor
ANOVA: Analysis of variance
CCD: Central composite design
CV: Coefficient of variation
DO: Dissolved oxygen
FAME: Fatty acid methyl ester
FFA: Free fatty acid
RSM: Response surface methodology
SCO: Single cell oil
SWW: Sago processing wastewater
YEME: Yeast extract malt extract

## References

[1] Dewangan A, Yadav AK, Mallick A (2018). Current scenario of biodiesel development in India: prospects and challenges. Energy Sources.

[2] Garay LA, Boundy-Mills KL, German JB (2014). Accumulation of high-value lipids in single-cell microorganisms: a mechanistic approach and future perspectives. J Agric Food Chem.

[3] Souza SP, Seabra JEA, Nogueira LAH (2018). Feedstocks for biodiesel production: Brazilian and global perspectives. Biofuels.

[4] Li C, Lesnik KL, Liu H (2013). Microbial conversion of waste glycerol from biodiesel production into value-added products. Energies.

[5] Matsakas L, Bonturi N, Miranda EA, Rova U, Christakopoulos P (2015). High concentrations of dried sorghum stalks as a biomass feedstock for single cell oil production by *Rhodosporidium toruloides*. Biotechnol Biofuels.

[6] Fontanille P, Kumar V, Christophe G, Nouaille R, Larroche C (2012). Bioconversion of volatile fatty acids into lipids by the oleaginous yeast *Yarrowia lipolytica*. Bioresour Technol.

[7] Ratledge C, Cohen Z (2008). Microbial and algal oils: do they have a future for biodiesel or as commodity oils?. Lipid Technol.

[8] Patel A, Arora N, Mehtani J, Pruthi V, Pruthi PA (2017). Assessment of fuel properties on the basis of fatty acid profiles of oleaginous yeast for potential biodiesel production. Renew Sustain Energy Rev.

[9] Poontawee W, Natakankitkul S, Wongmekiat O (2016). Protective effect of *Cleistocalyx nervosum* var. paniala fruit extract against oxidative renal damage caused by cadmium. Molecules.

[10] Wu J, Hu J, Zhao S, He M, Hu G, Ge X, Peng N (2018). Single-cell protein and xylitol production by a novel yeast strain *Candida intermedia* FL023 from lignocellulosic hydrolysates and xylose. Appl Biochem Biotechnol.

[11] Leiva-Candia DE, Pinzi S, Redel-Macías MD, Koutinas A, Webb C, Dorado MP (2014). The potential for agro-industrial waste utilization using oleaginous yeast for the production of biodiesel. Fuel.

[12] Rakicka M, Lazar Z, Dulermo T, Fickers P, Nicaud JM (2015). Lipid production by the oleaginous yeast Yarrowia lipolytica using industrial by-products under different culture conditions. Biotechnol Biofuels.

[13] Kouhia M, Holmberg H, Ahtila P (2015). Microalgae-utilizing biorefinery concept for pulp and paper industry: Converting secondary streams into value-added products. Algal Res.

[14] Sen B, Suttar RR (2012). Mesophilic fermentative hydrogen production from sago starch-processing wastewater using enriched mixed cultures. Int J Hydrogen Energy.

[15] Da Rós PCM, Silva GAM, Mendes AA, Santos JC, de Castro HF (2010). Evaluation of the catalytic properties of *Burkholderia cepacia* lipase immobilized on non-commercial matrices to be used in biodiesel synthesis from different feedstocks. Bioresour Technol.

[16] Ashika S, Kiruthika T, Ashokkumar K, Suraj H, Uthandi S (2017). Oleaginous Yeast from Sago waste water: screening and characterization of candida trophicalis for biolipid production. Madras Agric J.

[17] Thangavelu K, Sundararaju P, Srinivasan N, Muniraj I, Uthandi S (2020). Simultaneous lipid production for biodiesel feedstock and decontamination of sago processing wastewater using *Candida tropicalis* ASY2. Biotechnol Biofuels.

[18] Thangavelu K, Sundararaju P, Srinivasan N, Uthandi S (2021). Characterization of biomass produced by *Candida tropicalis* ASY2 grown using sago processing wastewater for bioenergy applications and its fuel properties. Biomass Conv Bioref.

[19] Srinivasan N, Thangavelu K, Sekar A, Uthandi S (2020). Characteristics of sago processing wastewater effluents released from different sago factories in Salem and Namakkal District of Tami Nadu India. Madras Agricultural Journal.

[20] Yen H-W, Liu YX (2014). Application of airlift bioreactor for the cultivation of aerobic oleaginous yeast *Rhodotorula glutinis* with different aeration rates. J Biosci Bioeng.

[21] Nantanga KK, Bertoft E, Seetharaman K (2013). Structures of human salivary amylase hydrolysates from starch processed at two water concentrations. Starch-Stärke.

[22] Tawil G, Jamme F, Réfrégiers M, Viksø-Nielsen A, Colonna P, Buléon A (2011). In situ tracking of enzymatic breakdown of starch granules by synchrotron UV fluorescence microscopy. Anal Chem.

[23] Thangavelu K, Sundararaju P, Uthandi S (2019). Rheology analysis of sago processing wastewater with variable starch content. Madras Agric J.

[24] Ren H-Y, Liu B-F, Kong F, Zhao L, Ren N (2015). Hydrogen and lipid production from starch wastewater by co-culture of anaerobic sludge and oleaginous microalgae with simultaneous COD, nitrogen and phosphorus removal. Water Res.

[25] Tanimura A, Takashima M, Sugita T, Endoh R, Kikukawa M, Yamaguchi S, Sakuradani E, Ogawa J, Ohkuma M, Shima J (2014). *Cryptococcus terricola* is a promising oleaginous yeast for biodiesel production from starch through consolidated bioprocessing. Sci Rep.

[26] Kraisintu P, Yongmanitchai W, Limtong S (2010). Selection and optimization for lipid production of a newly isolated oleaginous yeast, *Rhodosporidium toruloides* DMKU3-TK16. Kasetsart J (Nat Sci).

[27] Evans CT, Ratledge C (1984). Effect of nitrogen source on lipid accumulation in oleaginous yeasts. Microbiology.

[28] Minhas AK, Hodgson P, Barrow CJ, Adholeya A (2016). A review on the assessment of stress conditions for simultaneous production of microalgal lipids and carotenoids. Front Microbiol.

[29] Bandhu S, Dasgupta D, Akhter J, Kanaujia P, Suman SK, Agrawal D, Kaul S, Adhikari DK, Ghosh D (2014). Statistical design and optimization of single cell oil production from sugarcane bagasse hydrolysate by an oleaginous yeast *Rhodotorula* sp. IIP-33 using response surface methodology. SpringerPlus.

[30] Maran JP, Manikandan S, Nivetha CV, Dinesh R (2017). Ultrasound assisted extraction of bioactive compounds from *Nephelium lappaceum* L. fruit peel using central composite face centered response surface design. Arab J Chem.

[31] Mazaheri H, Lee KT, Bhatia S, Mohamed AR (2010). Subcritical water liquefaction of oil palm fruit press fiber for the production of bio-oil: effect of catalysts. Biores Technol.

[32] Guo W-Q, Ren N-Q, Wang X-J, Xiang W-S, Ding J, You Y, Liu B-F (2009). Optimization of culture conditions for hydrogen production by Ethanoligenens harbinense B49 using response surface methodology. Bioresour Technol.

[33] Liu J-Z, Weng L-P, Zhang Q-L, Xu H, Ji L-N (2003). Optimization of glucose oxidase production by *Aspergillus niger* in a benchtop bioreactor using response surface methodology. World J Microbiol Biotechnol.

[34] Yen H-W, Chang J-T, Chang J-S (2015). The growth of oleaginous *Rhodotorula glutinis* in an internal-loop airlift bioreactor by using mixture substrates of rice straw hydrolysate and crude glycerol. Biomass Bioenerg.

[35] Li M, Liu G-L, Chi Z, Chi Z-M (2010). Single cell oil production from hydrolysate of cassava starch by marine-derived yeast *Rhodotorula mucilaginosa* TJY15a. Biomass Bioenerg.

[36] Batista KA, Bataus LAM, Campos IT, Fernandes KF (2013). Development of culture medium using extruded bean as a nitrogen source for yeast growth. J Microbiol Methods.

[37] Milessi TS, Antunes FA, Chandel AK, Silva SS (2013). Rice bran extract: an inexpensive nitrogen source for the production of 2G ethanol from sugarcane bagasse hydrolysate. Biotech.

[38] Sridee W, Laopaiboon L, Jaisil P, Laopaiboon P (2011). The use of dried spent yeast as a low-cost nitrogen supplement in ethanol fermentation from sweet sorghum juice under very high gravity conditions. Electron J Biotechnol.

[39] Shaafi T, Velraj R (2015). Influence of alumina nanoparticles, ethanol and isopropanol blend as additive with diesel–soybean biodiesel blend fuel: Combustion, engine performance and emissions. Renew Energy.

[40] Sivalakshmi S, Balusamy T (2013). Effect of biodiesel and its blends with diethyl ether on the combustion, performance and emissions from a diesel engine. Fuel.

[41] Rangaswamy V, Saran S, Kannabiran M, Thiru M, Sankh S: Process for biodiesel production from a yeast strain. In *US Patent No 9,725,745 Washington, DC: US Patent and Trademark Office*: Google Patents; 2017.

[42] Papanikolaou S, Aggelis G (2011). Lipids of oleaginous yeasts. Part II: technology and potential applications. Eur J Lipid Sci Technol.

[43] Lotero E, Liu Y, Lopez DE, Suwannakarn K, Bruce DA, Goodwin JG (2005). Synthesis of biodiesel via acid catalysis. Ind Eng Chem Res.

[44] Van Gerpen J (2005). Biodiesel processing and production. Fuel Process Technol.

[45] Tiwari AK, Kumar A, Raheman H (2007). Biodiesel production from jatropha oil (*Jatropha curcas*) with high free fatty acids: an optimized process. Biomass Bioenerg.

[46] Kuwornoo DK, Ahiekpor JC (2010). Optimization of factors affecting the production of biodiesel from crude palm kernel oil and ethanol. Journal homepage: www IJEE IEEFoundation org.

[47] Leung DYC, Guo Y (2006). Transesterification of neat and used frying oil: optimization for biodiesel production. Fuel Process Technol.

[48] Lyoo W, Lee H (2002). Synthesis of high-molecular-weight poly (vinyl alcohol) with high yield by novel one-batch suspension polymerization of vinyl acetate and saponification. Colloid Polym Sci.

[49] Macías-Sánchez MD, Robles-Medina A, Hita-Peña E, Jiménez-Callejón MJ, Estéban-Cerdán L, González-Moreno PA, Molina-Grima E (2015). Biodiesel production from wet microalgal biomass by direct transesterification. Fuel.

[50] Thliveros P, Kiran EU, Webb C (2014). Microbial biodiesel production by direct methanolysis of oleaginous biomass. Bioresour Technol.

[51] Chopra J, Dineshkumar R, Bhaumik M, Dhanarajan G, Kumar R, Sen R (2016). Integrated in situ transesterification for improved biodiesel production from oleaginous yeast: a value proposition for possible industrial implication. RSC Adv.

[52] Eevera T, Rajendran K, Saradha S (2009). Biodiesel production process optimization and characterization to assess the suitability of the product for varied environmental conditions. Renew Energy.

[53] Alamu OJ, Waheed MA, Jekayinfa SO (2008). Effect of ethanol–palm kernel oil ratio on alkali-catalyzed biodiesel yield. Fuel.

[54] Ma F, Clements LD, Hanna MA (1998). The effects of catalyst, free fatty acids, and water on transesterification of beef tallow. Trans ASAE.

[55] Folch J, Lees M, Stanley GHS (1957). A simple method for the isolation and purification of total lipides from animal tissues. J Biol Chem.

[56] Atadashi I, Aroua MK, Aziz AA, Sulaiman N (2012). The effects of water on biodiesel production and refining technologies: a review. Renew Sustain Energy Rev.

[57] Sathish A, Smith BR, Sims RC (2014). Effect of moisture on in situ transesterification of microalgae for biodiesel production. J Chem Technol Biotechnol.

[58] Cheirsilp B, Louhasakul Y (2013). Industrial wastes as a promising renewable source for production of microbial lipid and direct transesterification of the lipid into biodiesel. Biores Technol.

[59] Martinez-Silveira A, Villarreal R, Garmendia G, Rufo C, Vero S (2019). Process conditions for a rapid in situ transesterification for biodiesel production from oleaginous yeasts. Electron J Biotechnol.

