# Supplementary material for: Bioconversion of sago processing wastewater into biodiesel: Optimization of lipid production by an oleaginous yeast, Candida tropicalis ASY2 and its transesterification process using response surface methodology
Source: Microb Cell Fact. 2021 Aug 26;20:167. doi: 10.1186/s12934-021-01655-7 (PMC8394618; doi:10.1186/s12934-021-01655-7)
Supplement: Supplementary file 1 — Additional file 1. Additional tables. [file 12934_2021_1655_MOESM1_ESM.docx]

**Supplementary Information**

**Table S1 ANOVA showing the effect of variables and their interactions for response biomass yield in ALB using CCD design**

| **Source** | **Sum of Squares** | **Degrees of freedom** | **Mean**  **Square** | ***F*-value** | ***p*-value** | **Significance** |
| --- | --- | --- | --- | --- | --- | --- |
| Model | 56.88 | 9 | 6.32 | 94.28 | < 0.0001 | S |
| *A*-Starch | 8.04 | 1 | 8.04 | 119.99 | < 0.0001 |  |
| *B*-Yeast extract | 2.42 | 1 | 2.42 | 36.03 | 0.0001 |  |
| *C*-Airflow rate | 38.20 | 1 | 38.2 | 569.89 | < 0.0001 |  |
| *AB* | 0.08 | 1 | 0.078 | 1.16 | 0.306 |  |
| *AC* | 0.53 | 1 | 0.5253 | 7.84 | 0.0188 |  |
| *BC* | 0.37 | 1 | 0.3741 | 5.58 | 0.0398 |  |
| *A²* | 5.31 | 1 | 5.31 | 79.18 | < 0.0001 |  |
| *B²* | 1.24 | 1 | 1.24 | 18.44 | 0.0016 |  |
| *C²* | 0.20 | 1 | 0.204 | 3.04 | 0.1116 |  |
| Residual | 0.67 | 10 | 0.067 |  |  |  |
| Lack of Fit | 0.25 | 5 | 0.0507 | 0.61 | 0.7002 | NS |
| Pure Error | 0.42 | 5 | 0.0833 |  |  |  |
| Cor Total | 57.55 | 19 |  |  |  |  |
| Std. Dev. | 0.26 | *R²* | | 0.99 |  |  |
| Mean | 5.13 | Adjusted *R²* | | 0.98 |  |  |
| C.V. % | 5.04 | Predicted *R²* | | 0.96 |  |  |
|  |  | Adeq Precision | | 33.58 |  |  |

**Table S2 ANOVA showing the effect of variables and their interactions for response lipid yield in ALB using CCD design**

| **Source** | **Sum of Squares** | **Degrees of freedom** | **Mean Square** | ***F*-value** | ***p*-value** | **Significance** |
| --- | --- | --- | --- | --- | --- | --- |
| Model | 9.45 | 9 | 1.05 | 48.75 | < 0.0001 | S |
| *A*-Starch | 2.78 | 1 | 2.78 | 128.97 | < 0.0001 |  |
| *B*-Yeast extract | 0.46 | 1 | 0.46 | 21.25 | 0.001 |  |
| *C*-Airflow rate | 1.16 | 1 | 1.16 | 53.80 | < 0.0001 |  |
| *AB* | 0.12 | 1 | 0.12 | 5.57 | 0.0399 |  |
| *AC* | 0.00 | 1 | 0.00 | 0.00 | 1 |  |
| *BC* | 0.14 | 1 | 0.14 | 6.52 | 0.0287 |  |
| *A²* | 2.26 | 1 | 2.26 | 105.00 | < 0.0001 |  |
| *B²* | 0.04 | 1 | 0.04 | 1.74 | 0.2161 |  |
| *C²* | 2.75 | 1 | 2.75 | 127.66 | < 0.0001 |  |
| Residual | 0.22 | 10 | 0.02 |  |  |  |
| Lack of Fit | 0.17 | 5 | 0.03 | 3.44 | 0.1008 | NS |
| Pure Error | 0.05 | 5 | 0.01 |  |  |  |
| Cor Total | 9.67 | 19 |  |  |  |  |
| Std. Dev. | 0.15 | *R²* | | 0.98 |  |  |
| Mean | 1.77 | Adjusted *R²* | | 0.96 |  |  |
| C.V. % | 8.28 | Predicted *R²* | | 0.85 |  |  |
|  |  | Adeq Precision | | 22.46 |  |  |

**Table S3 ANOVA showing the effect of variables and their interactions for response lipid content in ALB using CCD design**

| **Source** | **Sum of Squares** | **Degrees of freedom** | **Mean Square** | ***F*-value** | ***p*-value** | **Significance** |
| --- | --- | --- | --- | --- | --- | --- |
| Model | 2013.59 | 9 | 223.73 | 37.25 | < 0.0001 | S |
| *A*-Starch | 494.43 | 1 | 494.43 | 82.32 | < 0.0001 |  |
| *B*-Yeast extract | 400.31 | 1 | 400.31 | 66.65 | < 0.0001 |  |
| *C*-Airflow rate | 152.47 | 1 | 152.47 | 25.38 | 0.0005 |  |
| *AB* | 45.98 | 1 | 45.98 | 7.66 | 0.0199 |  |
| *AC* | 49.10 | 1 | 49.10 | 8.18 | 0.017 |  |
| *BC* | 28.12 | 1 | 28.12 | 4.68 | 0.0557 |  |
| *A²* | 357.81 | 1 | 357.81 | 59.57 | < 0.0001 |  |
| *B²* | 2.27 | 1 | 2.27 | 0.38 | 0.5521 |  |
| *C²* | 560.46 | 1 | 560.46 | 93.31 | < 0.0001 |  |
| Residual | 60.06 | 10 | 6.01 |  |  |  |
| Lack of Fit | 35.37 | 5 | 7.07 | 1.43 | 0.3515 | NS |
| Pure Error | 24.70 | 5 | 4.94 |  |  |  |
| Cor Total | 2073.66 | 19 |  |  |  |  |
| Std. Dev. | 2.45 | *R²* | | 0.97 |  |  |
| Mean | 34.59 | Adjusted *R²* | | 0.95 |  |  |
| C.V. % | 7.09 | Predicted *R²* | | 0.85 |  |  |
|  |  | Adeq Precision | | 18.58 |  |  |

**Table S4 ANOVA showing the effect of variables and their interactions for response FAME yield in the transesterification process using CCD design**

| **Source** | **Sum of Squares** | **Degrees of freedom** | **Mean Square** | ***F*-value** | ***p*-value** | **Significance** |
| --- | --- | --- | --- | --- | --- | --- |
| Model | 809.46 | 9 | 89.94 | 18.75 | < 0.0001 | S |
| *A*-Methanol | 482.19 | 1 | 482.19 | 100.51 | < 0.0001 |  |
| *B*-Catalyst  concentration | 80.12 | 1 | 80.12 | 16.70 | 0.0022 |  |
| *C*-Time | 0.31 | 1 | 0.31 | 0.06 | 0.8051 |  |
| *AB* | 2.40 | 1 | 2.40 | 0.50 | 0.4957 |  |
| *AC* | 24.99 | 1 | 24.99 | 5.21 | 0.0456 |  |
| *BC* | 2.29 | 1 | 2.29 | 0.48 | 0.5054 |  |
| *A²* | 114.37 | 1 | 114.37 | 23.84 | 0.0006 |  |
| *B²* | 6.86 | 1 | 6.86 | 1.43 | 0.2592 |  |
| *C²* | 101.68 | 1 | 101.68 | 21.19 | 0.001 |  |
| Residual | 47.98 | 10 | 4.80 |  |  |  |
| Lack of Fit | 29.75 | 5 | 5.95 | 1.63 | 0.3018 | NS |
| Pure Error | 18.22 | 5 | 3.64 |  |  |  |
| Cor Total | 857.44 | 19 |  |  |  |  |
| Std. Dev. | 2.19 | *R²* | | 0.94 |  |  |
| Mean | 76.33 | Adjusted *R²* | | 0.89 |  |  |
| C.V. % | 2.87 | Predicted *R²* | | 0.70 |  |  |
|  |  | Adeq Precision | | 15.49 |  |  |
